# Use and preferences regarding internet-based health care delivery in patients with chronic kidney disease

**DOI:** 10.1186/s12911-020-01375-9

**Published:** 2021-02-01

**Authors:** Lena Schiffer, Raoul Gertges, Mariel Nöhre, Elisabeth Schieffer, Uwe Tegtbur, Lars Pape, Martina de Zwaan, Mario Schiffer

**Affiliations:** 1 Department of Pediatric Kidney, Liver and Metabolic Diseases, Hannover Medical School, Carl Neuberg Str. 1, 30625 Hannover, Germany; 2 Department of Psychosomatic Medicine and Psychotherapy, Hannover Medical School, Hannover, Germany; 3 Department of Nephrology, University Hospital, Friedrich-Alexander University, Erlangen, Germany; 4 Project Kidney Transplantation 360°, Hannover Medical School, Hannover, Germany; 5 Department of Sports Medicine, Hannover Medical School, Hannover, Germany

**Keywords:** Chronic kidney disease (CKD), Video conferencing, eHealth, Internet-based technologies, Coronavirus disease 2019 (COVID-19)

## Abstract

**Background and objectives:**

Internet-based technologies play an increasingly important role in the management and outcome of patients with chronic kidney disease (CKD). The healthcare system is currently flooded with digital innovations and internet-based technologies as a consequence of the coronavirus disease 2019 (COVID-19) pandemic. However, information about the attitude of German CKD-patients with access to online tools towards the use of remote, internet-based interactions such as video conferencing, email, electronic medical records and apps in general and for health issues in particular, are missing.

**Design, setting, participants, and measurements:**

To address the use, habits and willingness of CKD patients in handling internet-based technologies we conducted a nationwide cross-sectional questionnaire survey in adults with CKD.

**Results:**

We used 380 questionnaires from adult CKD patients (47.6% on dialysis, 43.7% transplanted and 8.7% CKD before renal replacement therapy) for analysis. Of these 18.9% denied using the internet at all (nonusers). Nonusers were significantly older (74.4 years, SD 11.4) than users (54.5 years, SD 14.5, p < 0.001), had a lower educational level than users (≥ 12 years: 6.9% versus 47.1%, p < 0.001) and were more often on dialysis. Within the group of internet users only a minority (2.6%) was using video conferencing with their physician, only 11.7% stated that they were using email to report symptoms and 26.6% were using the internet to schedule appointments. Slightly more than one-third of internet users (35.1%) are concerned that their personal medical data are not safe when submitted via the internet.

**Conclusions:**

Within our group of German CKD-patients we found that almost one out of five patients, especially older patients and patients with a lower educational level, did not use the internet at all. The majority of internet users reported in our survey that they have not used internet-based technologies within a medical context so far, but are willing to consider it. Therefore, it seems to be important to introduce and teach motivated CKD-patients the use and benefits of simple and safe internet-based health care technologies.

## Background

The use of internet-based health care delivery tools is expanding worldwide [1]. The coronavirus disease 2019 (COVID-19) pandemic accelerated this development since internet-based health care services can help to minimize in-person care appointments and still maintain access to health-care, which is especially important for vulnerable patient groups [2–4]. Internet-based health care delivery is usually defined as the distribution of health-related services and information via internet technology and comprises in particular health care apps, electronic patient records and online video consultations. These technologies can improve the patient’s health and reduce costs for health care systems [5]. Moreover, it is well accepted that internet-based technologies can enhance patient autonomy and improve clinical outcome in a variety of common chronic diseases, such as chronic kidney disease (CKD), diabetes mellitus and asthma bronchiale [6, 7]. In addition, patients in medically underserved areas, patients with disabilities, who may have difficulties visiting a clinic, or patients with rare diseases who are dependent on highly specialized centers, benefit from the opportunities of internet-based interactions with their physicians which enables them to bridge distances [8].

In the US the provision of internet-based programs for patients on dialysis changed drastically. Before 2018, the dialysis patient needed to be in an approved originating site during the telehealth encounter, such as certain hospitals, doctor’s offices, hospital-based dialysis centers, and skilled nursing facilities. However, the Bipartisan Budget Act of 2018 included the home of a dialysis patient as an additional site of health care delivery for home dialysis patients providing more independence for CKD patients [9]. Important regulatory issues and financial reimbursements for services provided through internet-based technologies were recently also clarified in Germany and open the use for a broader application [10–12]. A variety of clinical trials that investigate and evaluate new remote, internet-based models regarding direct patient to caregiver interactions were recently financially supported by several governmental and non-governmental grants [13, 14]. The results of these studies can be expected in the near future. In general, these internet-based technologies for the exchange of knowledge place the patient in an active role and require the ability to use electronic devices. However, little is known about the habits and willingness of patients with chronic kidney disease (CKD) regarding the use of digital devices. CKD is a major public health concern with more than 70 million affected individuals worldwide and often leads to end-stage renal disease that requires renal replacement strategies such as dialysis or renal transplantation [15]. These are major cost drivers for health-care systems in developed countries [16–18]. Particularly for CKD-patients different clinical studies have shown that remote, internet-based management systems may improve the care of patients with advanced CKD, decrease the burden of time patients spend in medical visits and are clinically useful [19–22]. Initially, internet-based interventions were used at organizational levels (e.g. for information exchange between hospitals and doctors office) or to monitor dialysis, especially home dialysis outcomes. However, direct patient involvement in eHealth systems and patient-reported outcome measures based on internet technologies are increasing and are expected to improve outcomes and patient satisfaction [23].

Patients with CKD and end-stage renal disease are especially dependent on regular medical consultations due to their fragile and complex medical conditions. Close monitoring for disease progression and a coordinated transition to renal replacement therapy is essential. General parameters that are frequently assessed by the patient or his/her general practitioner, e.g. basic laboratory results, weight gain and blood pressure, are needed by the specialists for evaluation and initiation of further therapy. If these data can be transferred to the specialist remotely and without delay, there is, in many cases, no urge for a personal consultation by a nephrologist [19, 21, 22]. It has to be considered that patients with chronic diseases as CKD are often older and of a lower socioeconomic status [19, 24]. This is of interest since a lower socioeconomic status or older age are factors that contribute to differences in internet-access and lead to the so-called digital divide [19, 25]. We analyzed in this study for the first time the user status and the motivation of CKD patients to use internet-based technologies in Germany.

## Methods

### Recruitment

Self-report questionnaires were distributed to patients with CKD by their health-care providers, the magazine “the kidney patient” of the German patient association “Bundesverband Niere e.V.” and via the website of the innovation project NTx360° (www.ntx360grad.de). The participants in the study were allowed to submit the self-report questionnaire electronically or by mail. A prepaid envelope was provided. The response rate was 547 submissions. 380 were complete for age, gender, educational level, user status and therapy. Participants for this study had to be 18 years or older. All participants provided their written informed consent in accordance with the Helsinki declaration. The study was approved by the ethics committee of the Medical School of Hannover.

The following sociodemographic data were assessed: Sex (male and female), year of birth, years of school-education, occupational status (full-time, part-time, retired, disability pension, others). The following health-related determinants were assessed: time on dialysis (subdivided into patients 1–5 years, > 5 years and > 10 years on dialysis), kidney transplantation, and chronic kidney disease without the need for dialysis.

Participants were initially asked about how often they use the internet in general (never), sporadic (less than 1 day/week), frequently (1–3 days of the week), regularly (on 4–6 days of the week) and daily (every day).

Questions were chosen with the help of physicians of the Hannover Medical School who are involved in internet studies of CKD patients [13, 14] and were modified from the questionnaire study by *Paslakis *et al*.*, who examined in a nationwide study the use and preferences regarding internet health care delivery in the general German population [26]. The questions were short and target-oriented and can be found in the Additional file 1.

### Statistical analysis

Statistical analyses were performed using SPSS Statistics for Windows version 25.0 (IBM Corp.). Descriptive statistics (percentages, means and standard deviations) were calculated. Analyses of variance, T-tests and Chi-square tests for comparison between groups (users versus nonusers, males versus females, different treatment groups) and binary logistic regression analysis were conducted as appropriate. The level of significance was set at p ≤ 0.05.

## Results

### Cohort

A total of 380 data sets from individuals with CKD that were complete for age, gender, educational level, user status and therapy were used for analysis in this study. Patients from all federal states of Germany participated (Additional file 2: Figure S1).

The cohort consisted of 156 (41.1%) females and 224 (58.9%) males. Mean age was 64.5 years with a range from 18 to 93 years. Overall 181 (47.6%) reported to be on dialysis, 166 (43.7%) to be transplanted and 33 (8.7%) to suffer from CKD. More details on the sociodemographic information of the cohort are shown in Table 1.

**Table 1:** Comparison between male and female participants regarding sociodemographic factors

	Total	Males	Females	Statistics Chi-square
N (%)	380	224 (58.9%)	156 (41.1%)	
*Age categories (years)*				
18–34	35 (9.2%)	12 (5.4%)	23 (14.7%)	χ^2^ = 17.547 (df = 4), p = .002
35–44	38 (10%)	18 (8.0%)	20 (12.8%)	
45–54	58 (15.3%)	30 (13.4%)	28 (17.9%)	
55–64	125 (32.9%)	85 (37.9%)	40 (25.6%)	
65 +	124 (32.6%	79 (35.3%)	45 (28.8%)	
*Educational level*				χ^2^ = 0.008 (df = 1), p = 0.93
< 12 years	230 (60.5%)	136 (60.7%)	94 (60.3%)	
≥ 12 years	150 (39.5%)	88 (39.3%)	62 (38.7%)	
*Current occupation*				χ^2^ = 18.444 (df = 5), p = .002
Full-time	91 (23.9%)	63 (28.1%)	28 (18.2%)	
Part-time	50 (13.2%)	20 (8.9%)	30 (19.5%)	
Not working	13 (3.4%)	7 (3.1%)	6 (3.9%)	
Retired				
Old-age pension	125 (33.1%)	84 (37.5%)	41 (26.6%)	
Early retirement	76 (20.1%)	37 (16.5%)	39 (25.3%)	
Others	25 (6.6%)	13 (5.8%)	10 (6.5%)	
*Treatment group*				
Dialysis patients, 1–5 years	109 (28.7%)	67 (29.9%)	42 (26.9%)	χ^2^ = 4.993 (df = 4), p = 0.29
Dialysis > 5 years	40 (10.5%)	28 (12.5%)	12 (7.7%)	
Dialysis > 10 years	32 (8.4%)	19 (8.5%)	13 (8.3%)	
Transplant patients	166 (43.7%)	95 (42.4%)	71 (45.5%)	
CKD patients (no renal replacement therapy)	33 (8.7%)	16 (6.7%)	18 (11.5%)	

*CKD* chronic kidney disease

### Self-identified Internet users versus nonusers

When comparing internet users and nonusers, no difference in sex distribution was found. However, age, educational level and treatment of end-stage renal disease differed significantly between groups. Nonusers were significantly older (74.4 years, SD 11.4) than users (54.5 years, SD 14.5, p < 0.001) and had a lower educational level (< 12 years: 93.1% versus 52.9%, p < 0.001). A significant difference was found between nonusers and users regarding the rate of patients being on dialysis, being transplanted, and CKD patients without renal replacement therapy (p < 0.001). Dialysis patients were more often nonusers independent of the time of dialysis treatment (1–5 years, > 5 years or > 10 years). In contrast, transplant patients and CKD patients without renal replacement therapy reported more often to be internet users. Only 1 (1.4%) out of the 166 transplanted participants and 1 (1.4%) out of the 33 participants with CKD self-identified as nonusers. In Table 2 demographics of user groups are depicted, more details about the results concerning the willingness of adult internet users to consider the use of internet technologies within a medical context of consultation or treatment as well as their actual experiences with technologies of this kind within this specific context are shown in Tables 4 and 5. When we performed binary logistic regression analysis with the user status as the dependent variable, we found that treatment group and educational level remained significant predictors while user status was not entirely explained by age (Table 3).

**Table 2:** Comparison between user groups

	Internet users n = 308 (81.1%)	Internet non-users, n = 72 (18.9%)	Statistics
*Sex*			
Female	125 (40.6%)	31 (43.1%)	Χ^2^ = 0.147 (df = 1),
Male	183 (59.4%)	41 (56.9%)	p = 0.7
Age, mean (SD)	54.5 (14.5)	74.4 (11.4)	T = -11.016 (df = 378), p < 0.001
*Educational level*			
< 12 years	163 (52.9%)	67 (93.1%)	Χ^2^ = 39.342 (df = 1),
≥ 12 years	145 (47.1%)	5 (6.9%)	p < 0.001
*Treatment*			
Dialysis patients, 1–5 years	62 (20.1%)	47 (65.3%)	Χ^2^ = 91.758 (df = 4), p < 0.001
Dialysis > 5 years	26 (8.4%)	14 (19.4%)	
Dialysis > 10 years	23 (7.5%)	9 (12.5%)	
Transplant patients	165 (53.6%)	1 (1.4%)	
CKD patients (no renal replacement therapy)	32 (10.4%)	1 (1.4%)	

*CKD *chronic kidney disease

**Table 3:** Binary logistic regression analysis with user status as the dependent variable (users = 0, non-users = 1)

	Regression coefficient	Standard error	Wald	df	Sign	Odds ratio	95% CI Lower	95% CI Upper
Sex^1^	-0.529	0.409	1.673	1	0.196	0.589	0.264	1.313
Age	0.103	0.017	37.050	1	< 0.001	1.108	1.072	1.145
Treatment group^2^			19.271	4	0.001			
Dialysis 1–5 yrs	2.088	1.098	3.615	1	0.057	8.069	0.938	69.446
Dialysis > 5 yrs	2.386	1.147	4.327	1	0.038	10.875	1.148	103.045
Dialysis > 10 yrs	2.159	1.170	3.403	1	0.065	8.661	0.874	85.828
Transplantation	-1.882	1.470	1.640	1	0.200	0.152	0.009	2.713
Educational level^3^	2.090	0.559	14.000	1	< 0.001	8.088	2.706	24.177
Konstante	-10.730	1.618	43.959	1	< 0.001	0.000		

Reference category: ^1^female, ^2^CKD group, ^3^ ≥ 12 years of education

### Actual experience and willingness of patients to use internet, email and video conferencing within a medical context

When we asked the patients if they used the internet in general for work or for private purposes 72 (18.9%) denied using the internet at all (nonusers), while 308 patients (81.1%) reported to use the internet on a daily basis (Table 2).

Within the group of internet users 26.6% stated they have used email to schedule visits and 11.7% have used e-mail to report symptoms. Only 2.6% have used video conferencing with their physician, 3.6% have used electronic medical records and 2.9% have used apps. These findings are displayed in Table 4. In contrast 79.5% stated they would use email to schedule visits and 67.2% would use e-mail to report symptoms. 65.9% would use video conferencing with their physician, 73.4% would use electronic medical records and 75.6% would use apps. These findings are displayed in Table 5.

**Table 4:** Actual experiences of internet users (n = 308) with technologies of this kind within this specific context

	N (%)
Have used email to schedule visits	82 (26.6%)
Have used email to report symptoms	36 (11.7%)
Have used video conferencing with their physician	8 (2.6%)
Have used video conferencing with > 1 physician at the same time	4 (1.3%)
Have used electronic medical records	11 (3.6%)
Have used apps	9 (2.9%)

**Table 5:** Willingness of adult internet users (n = 308) to consider the use of internet technologies within a medical context of consultation or treatment and their actual experiences with technologies of this kind within this specific context

	Yes, would use, N (%)	No, would not use, N (%)	Missing, n
Would use email to schedule visits	245 (79.5%)	48 (15.6%)	15 (4.9%)
Would use email to report symptoms	207 (67.2%)	83 (26.9%)	18 (5.8%)
Would use video conferencing with their physician	203 (65.9%)	89 (28.9%)	16 (5.2%)
Would use video conferencing with > 1 physician at the same time	192 (62.3%)	97 (31.5%)	19 (6.2%)
Would use electronic medical records	226 (73.4%)	68 (22.1%)	14 (4.5%)
Would use apps	233 (75.6%)	63 (20.5%)	12 (3.9%)

### Use of the internet to receive or submit medical information

Within the group of internet users 68.2% stated that they are able to use the internet to receive information about health issues. Within the same group, 60.1% were convinced that they are able to distinguish between reliable and non-reliable sources. When asked if they used the internet to make decisions based on information on the internet 37.8% stated that they did. Concerns that personal medical data are not safe when submitted via the internet were stated by 35.4% of the internet users.

## Discussion

Our study reveals that the majority of CKD patients in Germany (81.1%) use the internet for general purposes, whereas 18.9% are nonusers. However, only a minority of users have experience with internet-based health care technologies. Different studies for the management and outcome of patients with chronic diseases, including patients with chronic kidney disease, imply that remote technologies may revolutionize the health care system regarding clinical outcome and cost reductions [27, 28]. In addition, mobile health apps are gaining popularity and more patients are supposed to use mobile technologies and informational websites to receive information about certain diseases and treatment opportunities [29–31]. It is also anticipated that a variety of new internet-based approaches will flood the market in the near future especially with adolescents and younger adults as the target group [32]. However, despite the fact that in our study not many CKD patients were using internet-based health care technologies, the majority of patients are willing to consider the use within a medical context of consultation or treatment. In particular they are willing to use email to schedule visits or report symptoms, to do video conferencing with their physician and to use electronic medical records and apps. This is of clinical importance since it is well accepted that these technologies improve the medical outcome of this vulnerable patient group [19]. The use and expansion of internet technologies for CKD patients that address health care issues are politically supported and play an increasing role in health care systems worldwide. Especially since the declaration of the coronavirus disease 2019 (COVID-19) outbreak by the World Health Organization on March 11, 2020 as a pandemic, internet-based technologies, particularly video consultations, have been promoted and scaled up to reduce the risk of transmission almost worldwide [3, 33, 34]. Videoconferences seem to be practical, cost-effective and of interest for the vulnerable CKD patients in times of pandemics [33]. In spite of the fact that almost all CKD patients in our study have not used videoconferences so far, 62.3% are willing to consider their use for medical purposes. At his point it remains unclear why the patients are not using videoconferences with their physicians yet. Most likely videoconferences were not offered to the patients, the patients were not aware of the option or they just did not know how to set up video conferencing systems.

Only 2.9% of the participants in our study have used medical apps. Of interest, the majority, 75.6% of the applicants, report that they would consider using apps for health issues in the future. In contrast, a recent survey conducted by *Paslakis *et al*.* revealed that only 46.7% of the general adult population in Germany would use apps that offer personalized information about their condition and give recommendation for exercises and support [26]. We explain this discrepancy with the fact that the participants in our cohort suffer from a chronic disease and thus have had to deal with health topics already in the past, whereas healthy individuals in the general population may have not. In a study by *Singh *et al*.* it was revealed that CKD patients’ impressions of quality of smart-phone apps were not directly linked to nephrologist ratings [35]. Guidelines and quality checks of internet related health resources may be therefore of significant relevance to ensure high standards for the consumers.

More internet users with CKD would consider the use of internet technologies to communicate with their physicians compared to the general German population [26]. Again, this may be due to the fact that CKD patients have frequent contact with their physician and are probably more aware of the benefits.

Well in line with other studies, the digital divide was also apparent in our study where, not surprisingly, nonusers were significantly older and had a lower educational level than the group of internet users. This is especially problematic, since statistically these patients suffer the most from a variety of different chronic diseases and would benefit the most from internet based technologies and health support [36]. In our study nonusers were more likely to be on dialysis as opposed to being transplanted. However, being on dialysis cannot be seen as an independent variable for being a nonuser, since our dialysis patients were significantly older (Table 6). This is well in line with the findings of *Taylor *et al*.* who described previously that patients with higher educational level and health literacy were more often selected for kidney transplantation [37].

**Table 6:** Median age per treatment group

Treatment	Age, years (SD)
*Treatment*	
Dialysis patients, 1–5 yrs	65.8 (16.3)
Dialysis > 5 years	62.2 (14.1)
Dialysis > 10 years	58.9 (13.5)
Transplant patients	52.9 (14.7)
CKD patients	53.2 (15.1)

The median age of the participants was 58 years

As mentioned above, a variety of studies suggest that there are benefits of internet-based technologies for health care issues. In this context, it has to be considered that in studies that analyze the benefits of eHealth systems for patient management, the participants were usually recruited from an already interested group of patients. *Ong *et al*.* therefore postulate that these studies may be prone to selection bias since patients without an affinity to internet-based technologies were presumably not included as participants [21]. Our study also has several limitations. In our set-up, it was possible for the participants to submit the questionnaire either via the internet or by using a prepaid regular mail envelope and both submission methods were used almost equally. However, it is possible that there are actually more non-users since non-users only had the mail-in option to submit the survey. Secondly, it is a national survey study with a limited number of participants who might not be representative of the German CKD population or CKD populations in other countries. Moreover, selection bias due to the distribution of the questionnaire and the exclusion of participants with incomplete questionnaire data is possible. Furthermore, we did not use a validated questionnaire but a series of questions generated by experts in nephrology who were also involved in the questionnaire design in the study by *Paslakis *et al., to make the results comparable to the findings in the general German population.

## Conclusions

The majority of internet-users in our survey reported that they would consider internet-based technologies within a medical context of consultation or treatment. The percentage of CKD patients willing to use internet-based health care delivery is higher than in the general German population. However, we found that almost 20% of CKD patients, especially older patients and patients with a lower educational level, did not use the internet at all. Therefore, it seems to be of great relevance to identify motivated CKD patients in order to introduce high-quality internet-based health care technologies to them. In addition, there should be a focus on outreach to elderly CKD patients and patients with a lower educational level. This concept could also provide a helpful blueprint for health systems in other countries. We believe that for the successful implementation of internet-based health care delivery for patients with chronic kidney disease, fully funded “teaching points” should be established and the effects should be assessed by clinical studies.

## Supplementary Information

**Additional file 1:** Supplementary information about the questionaire.

**Additional file 2: Figure S1**. Participant distribution in Germany based on postal code. Postal code analysis of patients revealed that patients from all over Germany participated in the survey. The majority of patients that participated were recruited in postal area 3 and 9 where the innovation fonds study ktx360° is actively running at Hannover Medical School, kidney transplant center Hann. Münden and University Hospital Erlangen (postal code area map modified from www.venue.de)

## Data Availability

Not applicable.

## Abbreviation

CKD: Chronic kidney disease
