# Supplementary material for: Use and preferences regarding internet-based health care delivery in patients with chronic kidney disease
Source: BMC Med Inform Decis Mak. 2021 Feb 1;21:34. doi: 10.1186/s12911-020-01375-9 (PMC7848877; doi:10.1186/s12911-020-01375-9)
Supplement: Supplementary file 1 — Additional file 1: Supplementary information about the questionaire. [file 12911_2020_1375_MOESM1_ESM.docx]

**Supplementary information about the questionaire**

To have the possibility to compare directly the results with the general population we used similar questions as *Paslakis et al*, who examined in a nationwide study the use and preferences regarding internet health care delivery in the general population (26).

1. Would you use/have you used email to: schedule visits with your physician? To report symptoms?

2. Would you use/have you used videoconferencing with your physician?

3. Would you use/have you used videoconferencing with more than one physician (e.g. general practitioner and specialist) at the same time?

4. Would you use/have you used videoconferencing for a diagnostic evaluation?

5. Would you use/have you used electronic medical records you can access at any time to see your exam results and leave messages?

6. Would you use/have you used an app that offers personalized information about your condition and would you use recommended exercises and support?

7. Do you consider yourself able to distinguish reliable from non-reliable medical information?

8. Does internet based information help you to make health care decisions?

9 Are you concerned that personal medical data are not safe when submitted via the internet?
